# Reinfection Dynamics of Disease-Free Cassava Plants in Three Agroecological Regions of Côte d’Ivoire

**DOI:** 10.3390/v17101393

**Published:** 2025-10-20

**Authors:** John Steven S. Seka, Justin S. Pita, Modeste K. Kouassi, William J. -L. Amoakon, Bekanvié S. M. Kouakou, Mariam Combala, Daniel H. Otron, Brice Sidoine Essis, Konan Evrard B. Dibi, Angela O. Eni, Nazaire K. Kouassi, Fidèle Tiendrébéogo

**Affiliations:** 1 Laboratoire d’Innovation Pour la Santé des Plantes, UFR Biosciences, Université Félix Houphouët-Boigny, Abidjan 22 BP 582, Côte d’Ivoire; 2 The Central and West African Virus Epidemiology (WAVE) for Food Security Program, Pôle Scientifique et D’innovation, Université Félix Houphouët-Boigny, Bingerville 22 BP 582, Côte d’Ivoire; 3 Centre National de Recherche Agronomique (CNRA), Bouaké 01 BP 1740, Côte d’Ivoire

**Keywords:** cassava, begomovirus, virus-free plantlets, reinfection dynamics, whiteflies, cassava mosaic disease

## Abstract

Cassava mosaic disease (CMD) is caused by begomoviruses and can result in yield losses of up to 90% in susceptible varieties. Using disease-free planting material from in vitro cultures is one of the most effective ways of controlling this disease. A CMD epidemiological assessment was conducted in fields established with disease-free plantlets in Bouaké, Dabou, and Man, selected for their contrasting agroecological and CMD prevalence conditions. Virus and whitefly species characterisation was performed using PCR and sequencing. CMD incidence and severity were lowest at the Man site and highest at the Dabou site. Although whitefly abundance was relatively low at the Man and Bouaké sites compared to the Dabou site, they were a significant factor in the spread of the disease. While all resistant varieties remained asymptomatic, susceptible and tolerant varieties became infected, and some tolerant varieties were able to recover from the disease. Molecular analyses revealed the presence of two viral species: *Begomovirus manihotis* (ACMV) and *Begomovirus manihotiscameroonense* (EACMCMV). No viral infection was detected 4 weeks after planting (WAP). Cases of single infection and double infection were observed at 12 and 20 WAP. Also, no double infections were found at the Man site, in contrast to the Bouaké site (12 WAP: 2.36%) and Dabou site (12 WAP: 2.59%; 20 WAP: 5.76%). EACMCMV was found in a single infection in Bouaké (12 WAP: 1.39%) and Man (20 WAP: 0.66%). The whitefly species *Bemisia tabaci* and *Bemisia afer* were most commonly found feeding on all cassava varieties. A high diversity of whitefly species was observed in Bouaké and Dabou compared to Man. Furthermore, the *Bemisia tabaci* species identified in this study was found to be able to transmit ACMV and EACMCMV viruses. These highlights would contribute to improving CMD management and control strategies.

## 1. Introduction

Cassava (*Manihot esculenta* Crantz) is a major source of carbohydrates for more than 800 million people across the world, particularly in tropical regions such as Africa, Asia, and Latin America [1]. However, cassava productivity in Africa remains relatively low, with major producers such as Nigeria and Côte d’Ivoire reporting yields ranging from 5.33 to 5.83 t/ha. This is low compared to other major cassava-growing regions, such as Thailand, which are reporting yields of up to 20.65 t/ha [2]. In Côte d’Ivoire, cassava is a major source of dietary energy and income, as it is cultivated across around 4/5 of the country. Its leaves and roots provide a variety of meals for local consumption, such as attiéké, foutou, and placali [3,4].

Cassava production is affected by several biotic and abiotic factors that significantly reduce its yield. Viral diseases are the most economically significant diseases that impact cassava production, with cassava mosaic disease (CMD) being the most common in Africa and causing up to 90% in yield losses [5,6]. This disease is caused by a complex of 11 Cassava mosaic begomoviruses (CMB), nine of which are present in Africa: *Begomovirus manihotis* (African cassava mosaic virus; ACMV), *Begomovirus manihotisafricaense* (East African cassava mosaic virus; EACMV), *Begomovirus warburgi* (South African cassava mosaic virus; SACMV), *Begomovirus manihotismalawiense* (African cassava mosaic Malawi virus; EACMMV), *Begomovirus manihotiscameroonense* (East African cassava mosaic Cameroon virus; EACMCMV), *Begomovirus manihotiszanzibarense* (East African cassava mosaic Zanzibar virus; EACMZV), *Begomovirus manihotiskenyaense* (East African cassava mosaic Kenya virus; EACMKV), *Begomovirus manihotismadagascarense* (Cassava Madagascar mosaic virus; CMMGV) and *Begomovirus manihotisburkinafasoense* (African cassava mosaic Burkina Faso virus; ACMBFV) [7]. Currently, ACMV and EACMCMV are the main begomoviruses present in cassava fields in Côte d’Ivoire [8,9,10,11]. These CMBs are spread both by infected cuttings and by the whitefly *Bemisia tabaci* [12,13].

In Côte d’Ivoire, Kouakou et al. [9] observed CMD across seven different agroecological zones, with disease incidence varying according to the fields and over time. Other authors have shown that some varieties are more susceptible to CMD than others [14,15]. The use of cassava cuttings from previous fields to establish new ones, regardless of their phytosanitary status, contributes to the spread of the disease. One solution to this problem is to produce large quantities of virus-free planting materials using meristem culture combined with thermotherapy [16,17,18]. This technique enhances plant regeneration, enabling plants to better express their agronomic potential [19]. The resulting in vitro plants are sanitised and ready for large-scale distribution to farmers. However, the pitfall is that, once in the farmers’ fields, these disease-free plants may become reinfected over time by CMD-infected whiteflies from neighbouring fields [5,20,21]. Furthermore, some varieties can lose their resistance after meristem culture and when transferred to the fields [22,23,24].

Several studies have been carried out on cassava in order to overcome constraints, including CMD epidemiology [8,9,11], field protocol development [25,26], variety improvement [27,28], sanitation of some varieties through meristem culture combined with thermotherapy [18], and in vitro micropropagation enhancement [29]. Although these studies have contributed to the improvement of agricultural techniques, their effectiveness could be significantly enhanced by the availability of healthy planting material. It is important to create and implement adequate conditions to achieve this objective. To date, very few studies have explored this reinfection dynamic in Côte d’Ivoire, and very little information is available on how long it takes for CMD-cleaned in vitro plantlets to become reinfected once established in an uncontrolled environment. Furthermore, high investment costs for quality planting material, resources, and technologies could limit research in this field. This study is the first to analyse the dynamics of CMD reinfection of healthy in vitro plantlets in Côte d’Ivoire.

CMD epidemiological studies have highlighted distinct conditions within the agroecological zone (AEZ). Bouaké, a locality in the centre (AEZ VI), has been identified as an area with mild viral pressure; Dabou, a locality in the south (AEZ I), as an area with high viral pressure; and Man, a locality in the west (AEZ III), as a very low-pressure area [8,9]. According to Akano et al. [30], these types of areas are very suitable for assessing the CMD spread dynamics. Therefore, this study aimed to improve our understanding of the CMD reinfection dynamics of cassava fields established with cleansed in vitro plantlets of 12 cassava varieties, with the ultimate goal of promoting the sustainable adoption of in vitro sanitised plantlets for producing and distributing healthy cassava planting material at scale in Côte d’Ivoire. This will help reduce the impact of CMD on cassava production in the country.

## 2. Materials and Methods

### 2.1. Biological Material

Twelve cassava varieties from in vitro plantlets were used as plant material in this study (Table 1). They were selected based on four characteristics: CMD resistance (resistant: 05, tolerant: 02, and susceptible: 05), yield (all varieties had a yield of at least 20 t/ha), the variety types (local or improved for disease/yield), and the quality of the tuber roots for transformation. The susceptible and tolerant plants were sanitised and propagated and weaned in the plant tissue culture laboratory of the Central and West African Virus Epidemiology (WAVE) Programme at Université Félix Houphouët-Boigny, as described by Seka et al. [29]. The absence of virus from the sanitised plant was confirmed by PCR using the protocol described by Yéo et al. [18].

### 2.2. Characteristics of the Study Sites and Experimental Design

**Figure 1:**
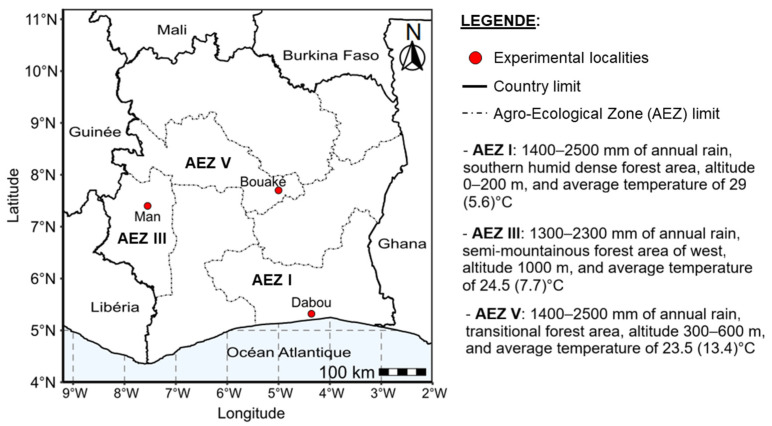
Map of Côte d’Ivoire highlighting the experimental sites as well as their respective agroecological zones.

The study was carried out simultaneously at three different sites (Bouaké, Dabou, and Man) in Côte d’Ivoire between June and December 2023 (Figure 1). These sites are characterised by abundant annual rainfall (an average of > 1500 mm per year) and an average annual temperature between 27 and 28 °C. The study sites were selected to ensure representativeness of different CMD incidences as previously reported by Amoakon et al. [8] and Kouakou et al. [9]. Dabou (5°19′ N, 4°23′ W) is located in agroecological zone I (AEZ I) and is characterised by fields with a high incidence of CMD (>75%). Bouaké (7°40′ N, 5°05′ W) is located in agroecological zone V (AEZ V) and is characterised by fields with a moderate incidence of CMD (25–50%). Man (7°20′ N, 7°36′ W) is located in agroecological zone III (AEZ III) is characterised by fields with a low incidence of CMD (<25%). A completely randomised design with three replications of twelve cassava varieties was used to conduct the experiments in this study. Each replication (block) was spaced two metres (m) apart and contained 12 plots, with one variety per plot arranged in rows. Each plot was 5 m long, with plants spaced 1 × 1 m apart. A total of 30 disease-free in vitro plantlets of each variety (360 in total per site) were transplanted to the field in June 2023 during the rainy season. Throughout the experiment, no fertiliser or pesticide was applied, and the fields were manually weeded as required.

### 2.3. Assessment of CMD Epidemiological Parameters

CMD epidemiological parameters were assessed between 2 weeks after planting (WAP) and up to 24 WAP. Leaves were visually assessed for the presence of characteristic CMD symptoms, such as leaf mosaic, chlorosis, leaf distortion, vein clearing, and stunted growth, and the whitefly population was evaluated by counting all adult whiteflies on the top five leaves of each plant. The mean of CMD symptom severity was calculated according to a scale of 1 to 5, with a score of 1 representing an absence of symptoms and scores of 2 to 5 representing increasing symptom severity, as described in Table 2 [31]. CMD incidence was calculated as the proportion of infected plants at a particular site [32]. As all plants were derived from in vitro cultures and certified virus-free by PCR, all CMD infections (mode of infection) observed were attributed to whitefly vectors. Data were collected using forms designed in the *KoboCollect* application (version 2023.2.4) and were downloaded from the *KoboCollect* server for subsequent analysis.

### 2.4. Sample Collection

Samples were collected after different epidemiological parameters were evaluated at 4, 12, and 20 weeks after planting (WAP). For asymptomatic cassava varieties, young leaves were collected from all plants, while for symptomatic varieties, samples were collected according to all levels of symptom severity. This sampling was carried out on the same plants during the experiment. These samples were kept in envelopes and stored at room temperature. Adult whiteflies were also collected on five young leaves according to the cassava variety and stored in 96% ethanol at −20 °C.

### 2.5. Molecular Analysis

#### 2.5.1. Total DNA Extraction and CMB Detection by Polymerase Chain Reaction (PCR)

Total DNA was extracted from 1011 cassava leaf samples according to the protocol described by Doyle and Doyle [33]. A Nanodrop (Eppendorf, Hamburg, Germany) was used to determine the concentration of each DNA extract, which was adjusted to 50 ng for use in polymerase chain reaction (PCR) amplification. PCR was performed using primer pairs specific for the detection of ACMV, EACMV, and EACMCMV (Table 3). The PCR master mix contained 5X Colourless GoTaq Reaction Buffer (Promega, Madison, WI, USA), 1 mM MgCl_2_ (Promega, USA), 0.2 mM dNTP (NEB), 0.4 μM of each primer (Eurogentec, Seraing, Belgium), and 0.625 U GoTaq polymerase (Promega, USA). DNA amplification was carried out using an Eppendorf Mastercycler X50 thermocycler with the following programme: an initial denaturation step at 94 °C for 4 min; followed by 35 cycles of 94 °C for 1 min, 55 °C for 1 min, and 72 °C for 1 min; and a final extension at 72 °C for 10 min. The PCR products (10 μL) were separated by 1% agarose gel electrophoresis, stained with ethidium bromide, and visualised under UV light using a gel imager.

#### 2.5.2. DNA Extraction and PCR Amplification of the Whiteflies’ Mitochondrial Cytochrome Oxidase I (mtCOI) Gene and CMBs Present in Field-Collected Whiteflies

Total DNA was extracted from individual adult whiteflies (216 in total) by placing them on a piece of parafilm placed on a Petri dish. Whitefly samples were ground in 60 µL of cold extraction buffer consisting of 5 µL of 1 M Tris-HCl (pH 8), 1 µL of 0.5 M EDTA (pH 8), 1 µL of proteinase K, 5 µL of Triton X-100, and 988 µL of ultrapure water. The mixture was then transferred to 0.6 mL Eppendorf tubes, and the tubes were incubated at 65 °C for 15 min, followed by 10 min at 95 °C. The tubes were then centrifuged at 10,000× *g* for 5 min, and the DNA was used for PCR amplification.

The mtCOI gene was amplified using the primer pair 2195Bt (5′-TGRTTTTTTGGTCATCCRCAAGT-3′) and C012/Bt-sh2 (5′-TTTACTGCACTTTCTGCC-3′) [37]. The reaction mixture contained Master Mix 2X (NEB), 10 µM of each primer, and 10 µL of DNA, giving a final volume of 50 µL. The PCR steps consisted of an initial denaturation step at 94 °C for 2 min; followed by 35 cycles of 94 °C for 30 s, 52 °C for 30 s, 72 °C for 1 min; and a final extension at 72 °C for 10 min. The PCR products were separated by electrophoresis in a 1% agarose gel stained with ethidium bromide. Amplification of CMBs present in whitefly samples was performed using 5 µL of DNA and the primers listed in Table 3. The reaction mixture preparation, PCR steps, and visualisation were carried out according to the protocols described above.

### 2.6. Sequencing and Phylogenetic Analysis

The PCR products from representative leaf and whitefly samples selected by the study site were sent to GENEWIZ^®^ (Leipzig, Germany) for Sanger sequencing. The raw sequences were cleaned and assembled using *Geneious Prime* software, version 2025.0.3. The consensus sequences obtained were then compared against the non-redundant database of NCBI using the BLASTn search utility. The sequences were then aligned separately with isolates representative of the diversity of cassava begomovirus isolates and whitefly species using the MEGA11 *Muscle* algorithms [38]. Maximum likelihood trees were constructed in MEGA11 using the nucleotide substitution model T92+G for ACMV and EACMCMV and the GTR+G model for whiteflies, with 1000 bootstrap replications. The most suitable substitution model was determined in advance using MEGA11. The phylogenetic trees were visualised and edited using FigTree version 1.4.3 (Edinburgh, UK).

### 2.7. Statistical Analysis

Generalised linear models (GLMs) were used to assess the site and variety effect (fixed effect) on CMD incidence, average CMD symptom severity, and whitefly abundance per plant. A GLM with a Poisson link function was used for CMD incidence, while GLMs with gamma link functions were used for symptom severity and whitefly abundance. When a significant site effect was detected, post hoc comparisons of estimated marginal means (emmeans function) were conducted using either Tukey or Sidak adjustments to control for type I error in multiple comparisons, depending on the model. Spearman’s rank was used to evaluate the correlation between whitefly abundance and both CMD symptom severity and disease incidence. To evaluate the site impact and whitefly abundance on infection rates, Kruskal–Wallis’s test was employed, followed by Wilcoxon rank-sum tests for post hoc comparisons. All statistical tests were conducted at a significance level of α = 0.05. Data visualisation, including maps and graphical summaries, was performed using the ggplot2 package in R [39]. All analyses were performed using various R packages (version 4.4.3; [40]).

## 3. Results

### 3.1. CMD Incidence

**Figure 2:**
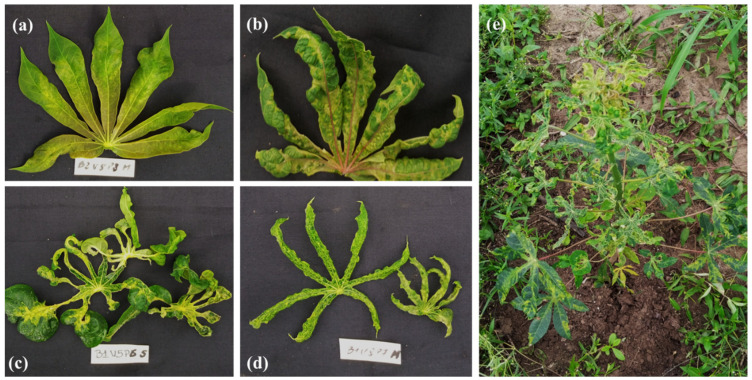
CMD symptoms observed at the experimental sites: (**a**) mild mosaic; (**b**) severe mosaic with moderate leaf deformation; (**c**) very severe mosaic with leaf deformation and curling; (**d**) filiform leaves; (**e**) complete leaf deformation and stunted growth of the entire plant.

**Figure 3:**
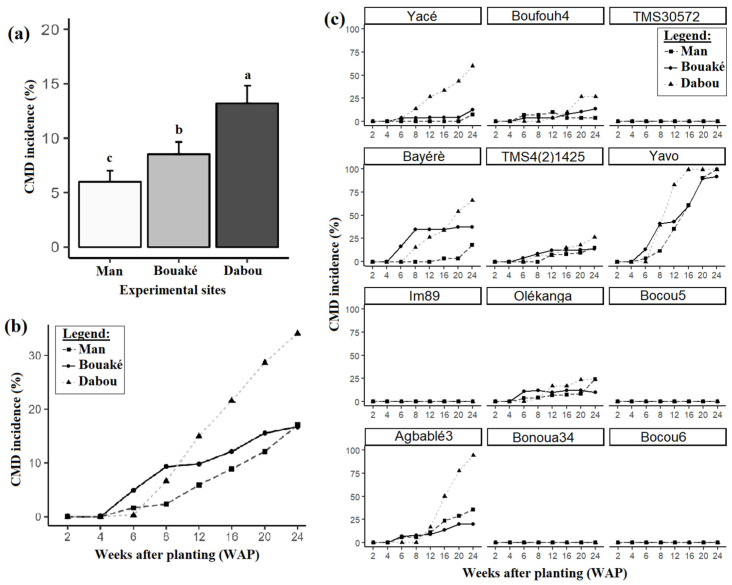
CMD incidence: (**a**) across the three experimental sites, (**b**) over time, and (**c**) across varieties. Data are presented as means ± standard error (SE). Error bars indicate SE. Site means (N = 340) sharing identical letters are not significantly different according to the Tukey-adjusted *emmeans* post hoc test at the 5% significance level.

CMD symptoms such as mild mosaic, severe mosaic, deformation of leaves, very severe mosaic and deformation, leaf curling, and filiform leaves were observed on all sites (Figure 2). The incidence of CMD varied significantly between sites. The lowest mean incidence of CMD was recorded at Man (5.9 ± 1.02%), while the highest mean incidence of CMD was recorded at Dabou (13.2 ± 1.62%; Figure 3a). The first symptoms were clearly visible as early as the sixth week after planting at the Bouaké and Man sites, whereas at the Dabou site, CMD symptoms were not observed until the eighth week (Figure 3b). CMD symptoms occurred at different times on the Agbablé 3 (V10), Bayèré (V4), Olékanga (V8), TMS4(2)1425 (V5), Yacé (V1), and Yavo (V6) varieties at the three sites. However, no symptoms were observed on plants from the following five varieties, irrespective of the sites: Bocou 5 (V9), Bocou 6 (V12), Bonoua 34 (V11), IM89 (V7), and TMS30572 (V3) (Figure 3c).

A significant site effect was also observed regarding the CMD incidence means between cassava varieties. Overall, the Dabou site showed the highest mean incidence. Varieties such as Yavo, Yacé, and Agbablé3 exhibited particularly pronounced contrasts across sites. However, Boufouh 4 (V2), Olékanga (V8), and TMS4(2)1425 (V5) showed low CMD incidence rates (less than 20%) at all three sites (Supplementary Table S1).

### 3.2. CMD Symptom Severity

**Figure 4:**
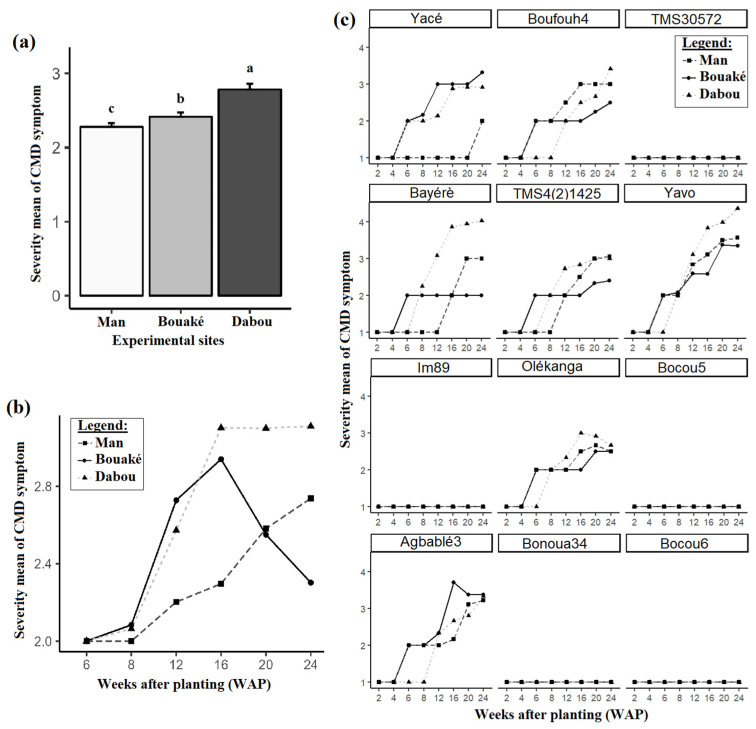
Severity means of CMD symptoms: (**a**) across the three experimental sites, (**b**) over time, and (**c**) across varieties. Data are presented as means ± standard error (SE). Error bars indicate SE. Site means (N = 340) sharing identical letters are not significantly different according to the Tukey-adjusted *emmeans* post hoc test at the 5% significance level.

The mean severity of CMD symptoms was relatively low (2–3) at all sites studied. However, the lowest mean disease severity was recorded in Man (2.36 ± 0.05), while the highest mean was recorded in Dabou (2.81 ± 0.08; Figure 4a). CMD symptom severity showed a gradual progression over time at the Dabou site compared to the Bouaké and Man sites (Figure 4b). The varieties Bayèré (V4) and Yavo (V6) exhibited more severe symptoms from the twelfth week (84 days after planting) following the transfer of the plantlets to the Dabou site (Figure 4c). The results indicate that the CMD mean severity is lower at the Man and Bouaké sites compared to the Dabou site for all the varieties except Yacé. The lowest mean was recorded for TMS4(2)1425 (V5). However, no symptoms were observed on the following five varieties: Bocou 5 (V9), Bocou 6 (V12), Bonoua 34 (V11), IM89 (V7), and TMS30572 (V3). These varieties showed no symptoms throughout the duration of the study at the three sites.

Statistical analysis showed significant differences in severity between Man, Bouaké, and Dabou sites for many varieties. In general, severity scores were higher in the Dabou site compared to other sites. Agbablé3, Bayèré, Boufouh4, Oléganga, Yacé, and Yavo exhibited significant variation, while TMS4(2)1425 showed no significant difference (Supplementary Table S1).

### 3.3. Mode of Infection, Whitefly Abundance per Plant, and Their Relationship with CMD Incidence and Severity of CMD Symptoms

**Figure 5:**
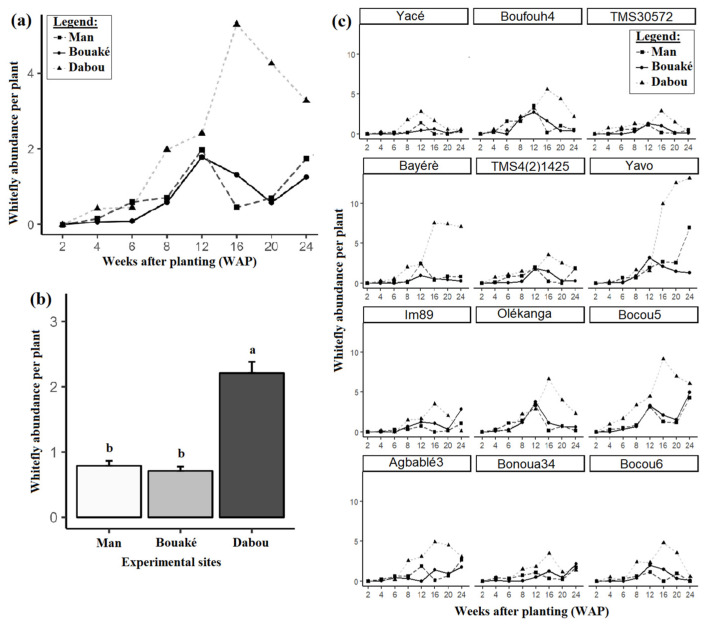
Whitefly abundance per plant at the three experimental sites: (**a**) across the three experimental sites, (**b**) over time, and (**c**) across varieties. Data are presented as means ± standard error (SE). Error bars indicate SE. Site means (N = 340) sharing identical letters are not significantly different according to the Tukey-adjusted *emmeans* post hoc test at the 5% significance level.

Our data indicate that the spread of CMD at the three sites (Bouaké, Dabou, and Man) might entirely be attributed to whitefly transmission. Although whiteflies were found to be the main cause of CMD propagation, their abundance per plant was relatively low at Man and Bouaké compared to Dabou throughout the 24-week observation period (Figure 5a). Whitefly abundance per plant was generally significantly higher in Dabou (2.25 ± 0.14) compared to the other two sites (0.79 ± 0.02 in Man and 0.71 ± 0.04 in Bouaké), as shown in Figure 5b. Notably, plants with higher whitefly counts (more than two individuals per plant) were observed predominantly on Bocou 5 (V9) and Yavo (V6) (Figure 5c).

Statistical analysis revealed significant differences in whitefly abundance across sites for most of the cassava varieties. The varieties Agbablé 3, Bayèré, Bocou 5, Bocou 6, Boufouh 4, Olékanga, TMS30572, TMS4(2)1425, Yacé, and Yavo showed the highest whitefly abundance in Dabou (see Supplementary Table S1). However, whiteflies were particularly abundant on the asymptomatic varieties V9 (Bocou 5), V12 (Bocou 6), V11 (Bonoua 34), V7 (IM89), and V3 (TMS30572).

Spearman’s correlation analysis showed a significant positive correlation (*p* < 0.05) between whitefly abundance per plant and CMD incidence (r = 0.46) at all three sites. Similarly, a significant positive correlation (*p* <0.05) was observed between whitefly abundance per plant and the severity of CMD symptoms (r = 0.41). The correlation between the incidence of CMD and whitefly abundance was stronger at the Man and Dabou sites (r = 0.52) compared to the Bouaké site (r = 0.1). Similarly, the correlation between the CMD severity and whitefly abundance was stronger at the Dabou (r = 0.55) and Man (r = 0.35) sites compared to the Bouaké site (r = 0.14) (Supplementary Figure S1).

### 3.4. Detection of ACMV and EACMCMV from Cassava Leaf Samples

A total of 1011 young cassava leaf samples were collected from the twelve varieties across the three experimental sites. Of these, 911 samples were asymptomatic (symptomless CMD) and 100 were symptomatic. PCR analysis revealed that 12.96% of the samples were infected with begomoviruses. The most prevalent virus was *Begomovirus manihotis* (ACMV), detected in a single infection in 9.89% of samples tested. *Begomovirus manihotiscameroonense* (EACMCMV) was detected in a single infection in only 0.3% of the samples tested. No *Begomovirus manihotisafricaense* (EACMV) infection was found. Mixed infections of ACMV and EACMCMV were found in 2.77% of the samples tested (Supplementary Table S2).

### 3.5. Presence of ACMV and/or EACMCMV, CMD Symptom Severity, and Plant Phytosanitary Status

Of the 100 samples showing CMD symptoms, 29 tested negative for all the primer pairs used. Approximately 58% of these symptomatic samples were infected by ACMV, while 23% were co-infected by ACMV and EACMCMV. EACMCMV was not detected in a single infection in all the symptomatic samples tested. Among the asymptomatic samples, CMB prevalence was 5.27%. ACMV was detected in 3.72% of the asymptomatic samples, while double infection of ACMV and EACMCMV was present in 0.88% of the samples. Additionally, 0.66% of the symptomless samples were infected with EACMCMV alone (Supplementary Table S3). CMD symptom severity varied according to the type of infection, as presented in Table 4. Samples with a single ACMV infection were more likely to show severe symptoms (53.66%) compared to samples with a mixed ACMV + EACMCMV infection, where symptoms were predominantly very severe (60%).

### 3.6. Correlation Between CMB Infection, Study Sites, and Whitefly Abundance

**Figure 6:**
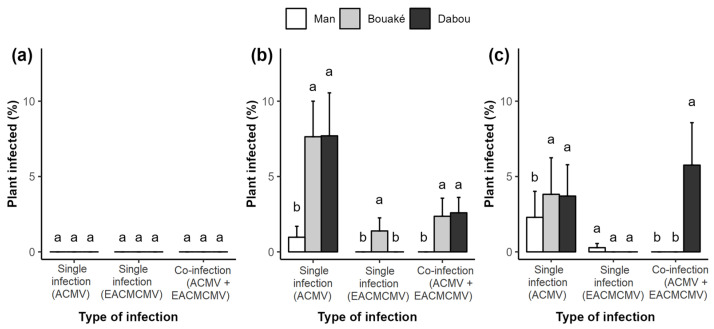
Percentage of cassava mosaic begomovirus (CMB) infections by experimental site at (**a**) 4 weeks, (**b**) 12 weeks, and (**c**) 20 weeks after planting. Data are presented as means ± standard error (SE). Error bars represent the SE. Bars with the same letter are not significantly different between sites for each infection type, based on Kruskal–Wallis and Wilcoxon paired non-parametric tests (*p* < 0.05).

The results indicate that the proportions of single ACMV infection, single EACMCMV infection, and double ACMV + EACMCMV infection varied across sites and depending on the data collection periods (Figure 6). No infections were detected at four weeks after planting (1 WAP) (Figure 6a). At 12 WAP, we observed ACMV single infections with a significantly higher proportion at Bouaké (7.64%) and Dabou (7.7%) compared to Man (0.97%). EACMCMV single infections were only detected in Bouaké, with a prevalence of 1.39%. Regarding ACMV + EACMCMV co-infections, the proportions were significantly higher in Bouaké (2.36%) and Dabou (2.59%). Overall, the proportion of ACMV single infections was higher than that of the other two infection types across all sites (Figure 6b). At 20 WAP, the proportion of ACMV infection alone increased, and it was higher at the Bouaké (3.82%) and Dabou (3.7%) sites than at the Man site (2.29%), where the proportion decreased. The Man site recorded EACMCMV single infection for the first time, and it made up the highest proportion. Only the Dabou site had an ACMV + EACMCMV co-infection, with an increased proportion of 5.76% (Figure 6c).

**Figure 7:**
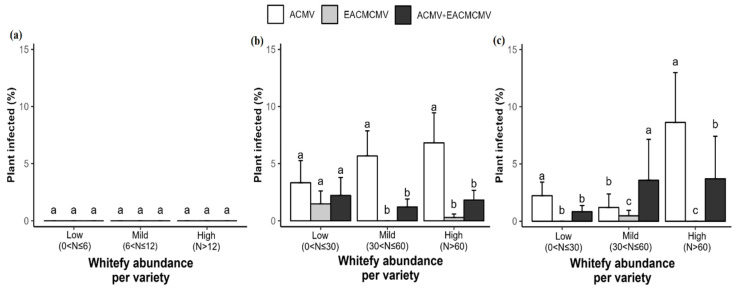
Percentage of cassava mosaic begomovirus (CMB) infections by whitefly abundance per variety at (**a**) 4 weeks, (**b**) 12 weeks, and (**c**) 20 weeks after planting. Data are presented as means ± standard error (SE). Error bars represent the SE. Bars with the same letter are not significantly different between sites for each infection type, based on Kruskal–Wallis and Wilcoxon paired non-parametric tests (*p* < 0.05).

The abundance of whiteflies by variety was correlated with the type of viral infection observed in the plants (Figure 7). Four weeks after planting (WAP), no infection was detected, regardless of whitefly abundance (Figure 7a). At 12 WAP, no significant differences in infection types were observed for varieties with low whitefly populations. For varieties with high whitefly abundance (6–12 whiteflies), single ACMV infections (5.67%) were more prevalent than ACMV + EACMCMV co-infections (1.21%), with no EACMCMV single infections recorded. For varieties with very high whitefly populations, the proportion of ACMV single infections increased to 6.82%, followed by co-infections (1.82%) and single EACMCMV infections (0.3%) (Figure 7b). At 20 WAP, single ACMV infections were again more prevalent (2.23%) for varieties with low whitefly abundance, but their proportion decreased compared to 12 WAP. For varieties with moderate whitefly populations, co-infections were more frequent (3.57%), whereas single EACMCMV infections were less common (0.48%). In varieties with a very high whitefly population, single ACMV infections peaked at 8.62%, followed by mixed infection (3.7%). These proportions increased in contrast to 12 WAP (Figure 7c).

### 3.7. Type of Infection and Level of CMD Resistance of Different Varieties

Of the 12 varieties evaluated in this study, 5 were susceptible, 2 were tolerant, and 5 were resistant to CMD. Molecular analyses revealed that the dynamics of single infection by ACMV and EACMCMV, as well as co-infection by ACMV and EACMCMV, varied according to the level of resistance of the evaluated varieties (Supplementary Figure S2).

Only ACMV infection was detected in susceptible varieties (Agbablé3, Bayérè, TMS4(2)1425), Yacé, and Yavo) in the Man site at 12 WAP (2.33%) and 20 WAP (4.83%). At the Bouaké site, all three infection types were observed at 12 WAP, with a high incidence of single ACMV infection (12%). However, at 20 WAP, only ACMV infection was detected (8.5%). At the Dabou site, all infection types were also present at 12 WAP, with ACMV being the most prevalent (13.15%). At 20 WAP, the incidence of ACMV decreased (7.96%), while the rate of co-infection with EACMCMV increased (8.89%).

For the tolerant varieties (Boufouh4 and Olékanga), only ACMV single infection was detected in the Man site at both 12 and 20 WAP, with a similar infection rate at both times. At the Bouaké site, data from 12 WAP showed the highest infection rate for ACMV single infection (15.83%), compared to EACMCMV single infection (1.67%) and ACMV + EACMCMV co-infection (1.08%). At 20 WAP, the infection rate for both EACMCMV single infection and co-infection fell sharply to 0%. In the Dabou site, the incidence of the mixed infection ACMV + EACMCMV increased markedly from 5.83% at 12 WAP to 14.17% at 20 WAP, while the incidence of single ACMV infection decreased from 14,67% to 4.17%.

Out of the five resistant varieties (Bocou 5, Bocou 6, Bonoua 34, IM89, and TMS30572), only one (TMS30572) was infected by EACMCMV in a single infection at the Man site, with an incidence rate of 0.67%. In summary, the Man site recorded ACMV single infection in both susceptible and tolerant varieties, whereas resistant varieties showed little EACMCMV infection. At the Bouaké site, all the resistant varieties remained virus-free, whereas susceptible and tolerant varieties were infected by the three virus combinations. In the Dabou site, resistant varieties remained infection-free, while susceptible and tolerant varieties displayed single ACMV infections and ACMV + EACMCMV co-infections (Supplementary Figure S2).

### 3.8. Phylogeny of the CMBs Detected in the Tested Leaf Samples

**Figure 8:**
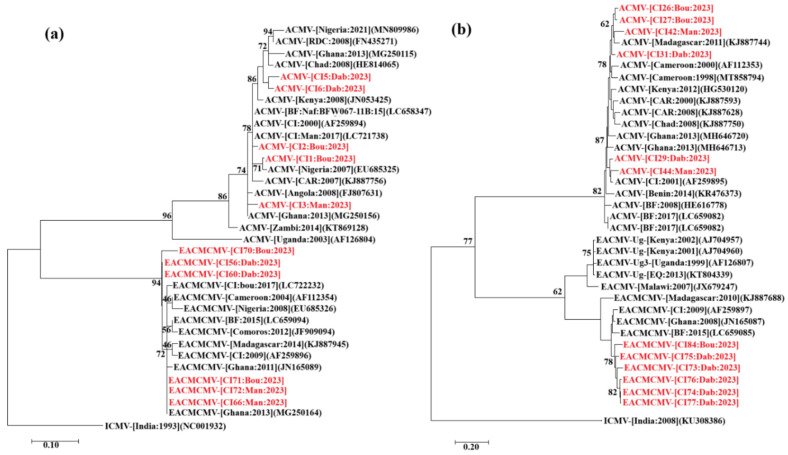
Maximum likelihood phylogenetic trees showing the relationships between Côte d’Ivoire isolates of *Begomovirus manihotiscameroonense* (EACMCMV; six isolates) and *Begomovirus manihotis* (ACMV; six isolates) alongside representative cassava mosaic begomovirus (CMB) isolates from GenBank. (**a**) Phylogenetic tree based on partial DNA-A sequences of EACMCMV and ACMV, rooted with *Begomovirus manihotisindianense* (ICMV) DNA-A (GenBank accession: NC001932) as the outgroup. (**b**) Phylogenetic tree based on partial DNA-B sequences of EACMCMV and ACMV, rooted with ICMV DNA-B (GenBank accession: KU308386) as the outgroup. Sequences obtained in this study are shown in red; reference sequences from GenBank are shown in black. Bootstrap analysis was conducted with 1000 replicates.

A total of 76 high-quality nucleotide sequences were obtained from direct sequencing of PCR amplicons. These included 13 sequences from Man, 15 from Bouaké, and 48 from Dabou. The sequences corresponded to 21 DNA fragments covering the AV1/AC3 genes of ACMV DNA-A, 26 fragments covering the BV1/BC1 genes of ACMV DNA-B, 17 covering the AC2/AC3 genes of EACMCMV DNA-A, and 12 DNA fragments covering the BC1 gene of EACMCMV DNA-B. BLASTn comparisons with sequences in the GenBank database revealed that nine of the sequences covering the AV1/AC3 genes were closely related to the ACMV isolates from Burkina Faso (LC658963, LC658347, and LC658961), with nucleotide identities ranging from 96% to 98.89%. Five sequences were more closely related to the ACMV isolates from Nigeria (EU685318, X17095, and MN809986), with nucleotide identity between 94.26% and 96.89%. One AV1/AC3 gene sequence showed close similarity to an ACMV isolate from Ghana (MG250152), with 97.96% nucleotide identity. Additionally, six sequences were closely related to ACMV isolates from Côte d’Ivoire (LC723865, LC721738, and LC721740), displaying nucleotide identities ranging from 96.82% to 99.75%. Among the BV1/BC1 gene sequences of ACMV DNA-B, one sequence was closely related (93.89%) to a Nigerian isolate (X17096), while 17 sequences were closely related to the Ghanaian isolates (MH646721, MH646723, and MH646698), sharing 96.29% to 98% nucleotide identity. Eight sequences matched ACMV DNA-B isolates from Côte d’Ivoire (LC724016, LC723866, LC722237, and AF259895), with nucleotide identities ranging from 94.4% to 99.11%. For the AC2/AC3 genes of EACMCMV DNA-A, seven sequences showed high similarity to Nigerian isolates (EU685326 and EU685323), with nucleotide identities between 95.76% and 98.86%. Four sequences of the AC2/AC3 genes were closely related to a Ghanaian isolate (JN165089), with nucleotide identities ranging from 95.57% to 99.11%. Six additional sequences showed high similarity (95.32% to 99.38%) to isolates from Côte d’Ivoire (LC722231, LC722232, and LC722234). All the sequences of the BC1 gene from EACMCMV DNA-B exhibited close relationships with a Ghanaian isolate (JN165087), sharing nucleotide identities between 91% and 95.63%. A maximum likelihood phylogenetic analysis, based on alignment of the sequences obtained in this study, revealed strong genetic clustering with isolates from Burkina Faso, Côte d’Ivoire, Ghana, and Nigeria (Figure 8a,b).

### 3.9. Identification of Whitefly Species and Characterisation of the CMBs They Carry

**Figure 9:**
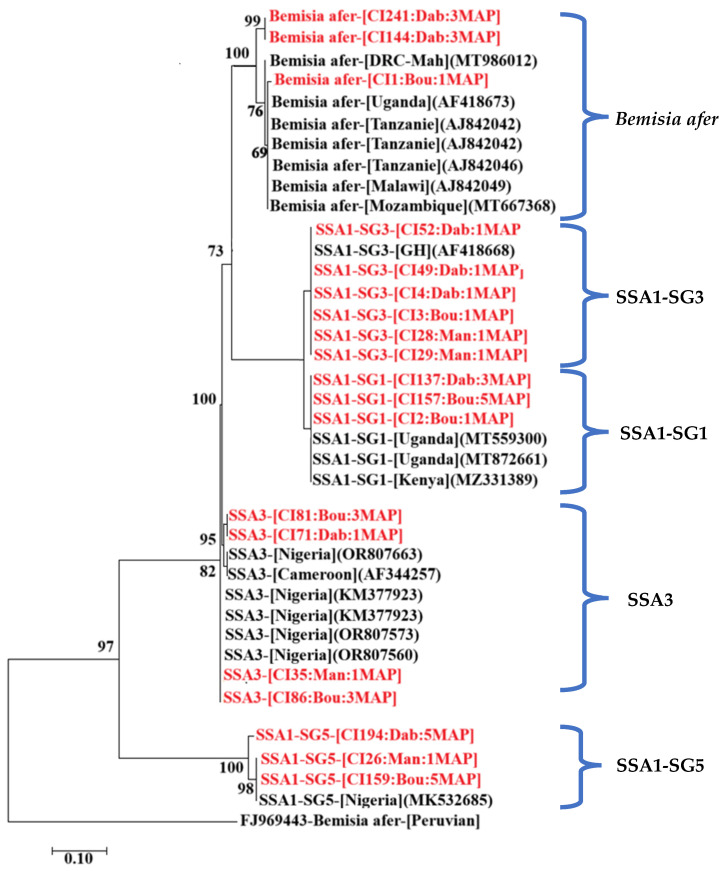
Phylogenetic tree of whitefly isolates collected at the study sites, based on partial mtCOI gene sequences. The tree was rooted using *Bemisia afer* (GenBank accession, FJ969443). Sequences obtained in this study are in red, while those in black were taken from GenBank. Bootstrap analysis was performed with 1000 replicates.

A total of 212 whitefly PCR amplicons were obtained with the mtCOI primers at all sites (Bouaké, Dabou, and Man). Sequencing of these amplicons generated 109 high-quality sequences: 46 from Bouaké, 41 from Dabou, and 22 from Man. Two whitefly species were identified from the sequence analysis: *Bemisia tabaci* (SSA1-SG1, SSA1-SG3, SSA1-SG5, and SSA3) and *Bemisia afer*. *Bemisia afer* was found only at the Bouaké and Dabou sites. In Man, the *B. tabaci* sequences obtained were closely related to the SSA1-SG3, SSA1-SG5, and SSA3 biotypes at 4 WAP and to a single biotype (SSA1-SG3) at 12 and 20 WAP. However, different biotypes of *Bemisia tabaci* were identified at the Bouaké site (four biotypes: SSA1-SG1, SSA1-SG3, SSA1-SG5, and SSA3) and Dabou (three biotypes: SSA1-SG3, SSA1-SG5, and SSA3) over time. The phylogenetic analysis, which clustered into five distinct groups (SSA1-SG1, SSA1-SG3, SSA1-SG5, SSA3, and *Bemisia afer*), supported these findings further (Figure 9).

PCR and sequencing revealed the presence of two begomovirus species in the whiteflies tested: *Begomovirus manihotis* (ACMV) and *Begomovirus manihotiscameroonense* (EACMCMV). A total of 216 whiteflies (72 for each site, Bouaké, Dabou, and Man) were tested for the presence of the viruses. The overall detection rate across all sites was 93.52%. Co-infection by ACMV and EACMCMV was more prevalent than single infections, with a detection rate of 63.43% (137/216), 14.35% (31/216) for ACMV, and 15.74% (34/216) for EACMCMV.

## 4. Discussion

Cassava mosaic disease (CMD) is the most devastating cassava disease in West Africa, causing annual economic losses exceeding US $2.7 billion [41]. CMD can reduce yields by up to 90% in susceptible varieties and by up to 50% in tolerant varieties [5]. CMD-related losses have an immediate impact on food supply and threaten the food security of the rapidly growing African populations [42]. CMD is transmitted by the whitefly (*Bemisia tabaci*) and is also spread by human activities, primarily through the trade and use of infected cassava cuttings to establish new fields [41]. Recent studies conducted in Côte d’Ivoire by Amoakon et al. [8] and Kouakou et al. [9] have demonstrated that over 90% of the cassava cuttings used as planting materials are infected with CMD. Moreover, continuous use of infected cuttings could provoke viral recombination and/or reassortment and increase the risk of a severe CMD epidemic.

This highlights how crucial the use of disease-free cuttings is for preventing potential CMD outbreaks and their adverse effects on cassava production [43]. In this context, large-scale production and distribution of clean planting materials (plants sanitised by in vitro culture or disease-free cuttings from previously sanitised plants) is imperative. However, susceptible varieties are at risk of reinfection by whitefly vectors (*Bemisia tabaci*) once transferred to the field [5]. Furthermore, the in vitro culture technique applied to tolerant or resistant varieties can result in the loss of the CMD resistance [24]. Therefore, to generate scientific evidence to guide cassava farmers, stakeholders, and decision-makers on the best use of cassava disease-free planting materials, we conducted this study on the reinfection dynamics of previously sanitised cassava plants in three localities in Côte d’Ivoire.

The epidemiological assessments conducted at different sites of three different agroecological regions revealed considerable variation in disease characteristics during 24 weeks (6 months), according to Sseruwagi et al. [32]. These authors indicated that the optimal period for CMD symptom development is 2 to 6 months. The Man site showed the lowest mean incidence and severity, while the Bouaké site was intermediate. The Dabou site presented the highest mean values for both disease incidence and disease severity. These differences could be attributed to the difference in altitude between the three agroecological regions (Man: 1060 m; Bouaké: 399 m; Dabou: 15 m), suggesting that CMD progresses differently at various altitudes, as demonstrated by Amoakon et al. in Côte d’Ivoire [8] and Harimalala et al. in Madagascar [44]. At higher altitudes, the disease develops more slowly, and symptoms tend to be less severe. In contrast, at lower altitudes, the disease progresses rapidly, and symptoms are more pronounced. Although there were relatively fewer whiteflies at the Man and Bouaké sites compared to the Dabou site, we found whiteflies to be the main vectors of disease spread at the three locations. This suggests that the whitefly species present in cassava in Côte d’Ivoire might be highly effective at spreading CMD. Furthermore, a strong positive correlation was observed between the whitefly abundance per plant, CMD incidence, and the severity of CMD symptoms. These findings emphasise the significant role played by whiteflies in CMD epidemiology in Côte d’Ivoire.

Indeed, in this study, the first CMD symptoms were observed six weeks after planting (6 WAP) in fields established with disease-free cassava plantlets, revealing the preponderant role whiteflies might play in spreading the disease in Côte d’Ivoire. This was not captured in the studies conducted by Amoakon et al. [8] and Kouakou et al. [9], which concluded that the main mode of CMD propagation in Côte d’Ivoire is through infected cuttings. Whiteflies’ impact on CMD transmission was imperceptible in these studies, probably because, unlike our experiments, their work was conducted in farmers’ fields, which are usually established with cassava cuttings that are already infected with CMD.

During our study, the abundance of whiteflies per plant increased significantly between the sixth and twelfth weeks after planting at the three studied sites. After the twelfth week, a significant reduction in whitefly population was observed at the Man and Bouaké sites. In contrast, whitefly populations at Dabou continued to increase until week 16 before decreasing significantly. The notable surge in whitefly numbers observed between weeks six and twelve at the Man and Bouaké sites, as well as between weeks six and sixteen at the Dabou site, may be associated with the rapid plant growth observed during this period. Indeed, during this time, the high number of new leaves on the young plants likely attracted high numbers of whiteflies, as noted by Zinga et al. [6]. The decline in whitefly populations observed later may be attributed to the rainy season. Indeed, after the twelfth week, heavy and frequent rains were recorded at the study sites.

Regarding whitefly attractiveness, two varieties, Bocou 5 and Yavo, were very distinct from the other 10 varieties studied due to the elevated populations of whiteflies feeding on them. Variation in whitefly attractiveness between different varieties can be attributed to the distinct intrinsic characteristics of each variety. Some varieties release specific volatile organic compounds that attract whiteflies, acting as olfactory signals that guide the insects toward the plant, whereas other varieties may produce secondary compounds, such as cyanoglucosides or phenols, which possess repellent properties against whiteflies [45]. Additionally, factors like leaf structure, particularly the presence of pubescent (hairy) leaves, may influence whitefly attractiveness. Previous studies have reported positive correlations between the presence of *Bemisia tabaci* and highly pubescent leaves in various crops [45,46,47].

Finally, whitefly abundance per plant was significantly higher at Dabou compared to Man and Bouaké. This difference is likely due to Dabou’s lower altitude, as *B. tabaci* has difficulty adapting to high altitudes (above 1000 m), as indicated by Morales and Jones [48] and the high incidence of CMD observed in Dabou [9].

This study confirmed the presence of *Begomovirus manihotis* (ACMV) and *Begomovirus manihotiscameroonense* (EACMCMV) in single and co-infections in the samples tested. Interestingly, no *Begomovirus manihotisafricaense* (EACMV) was observed. These findings align with those of Kouakou et al. [9], who reported similar observations in Côte d’Ivoire. Our work confirmed the resistance/susceptibility status of the 12 cassava varieties studied, which included 5 susceptible, 2 tolerant, and 5 resistant. The susceptible varieties (Agbablé3, Bayérè, TMS4(2)1425, Yacé, and Yavo) showed severe CMD symptoms, with infection rates reaching up to 100%. These varieties were infected by both ACMV and EACMCMV in single and co-infections. The tolerant varieties (Boufouh4 and Olékanga) displayed moderate CMD symptoms with low infection rates (less than 20%) and were infected by ACMV and EACMCMV in both single and co-infections. The resistant varieties (Bocou 6, Bocou 5, Bonoua 34, IM89, and TMS30572) showed no visible CMD symptoms at the three study sites. However, molecular analysis revealed that the variety TMS30572 was infected by EACMCMV in a single infection. This finding suggests that TMS30572 may not be resistant to CMD but rather a tolerant variety. It is also possible that TMS30572 lost its resistance via the meristematic culture we used for variety sanitation in this study. According to Chauhan et al. [24], the loss of CMD resistance is primarily due to disruptions in meristem integrity. If this was the case for variety TMS30572, then such disruption probably did not occur for the other four resistant varieties (Bocou 5, Bonoua 34, Bocou 6, and IM89), which remained uninfected throughout the duration of our study. Therefore, these four varieties are strong candidates for large-scale deployment across Côte d’Ivoire to contribute to CMD management. Amoakon et al. [14] also reported these varieties to be effective against CMD. Importantly, their resistance status remained stable even after undergoing meristem culture regeneration, indicating that genetic transformation and modification can be applied to these cultivars without compromising their CMD resistance.

A drastic reduction (down to 0%) in single EACMCMV infection and ACMV + EACMCMV co-infection rate was observed for the tolerant varieties. This significant reduction in viral infections can be explained by the plant’s natural recovery process, known as remission. In this context, remission refers to the spontaneous improvement in the phytosanitary condition of plants infected by one or more viruses. The primary mechanism underlying this process is gene silencing, particularly post-transcriptional gene silencing (PTGS), also known as RNA-mediated virus resistance [49,50,51]. PTGS involves the recognition and specific degradation of viral messenger RNA (mRNA), thereby inhibiting its translation. This results in a reduction in viral titre and/or the disappearance of symptoms in new leaves [52]. Although geminiviruses, such as ACMV and EACMCMV, do not produce dsRNA during their life cycle, they can still induce PTGS by generating virus-derived siRNAs [53]. However, at the Dabou site, no signs of remission were observed. Instead, there was a significant increase in the rate of ACMV + EACMCMV co-infection, despite a decrease in the rate of ACMV single infections. These findings suggest two possibilities: (1) the viral strains present in Dabou may express a potent viral suppressor of RNA silencing because of the very elevated CMD pressure and elevated whitefly populations existing in this site; or (2) the plant’s apoptosis pathway may have been prematurely activated, interfering with the PTGS process [53].

It is important to specify that some symptomatic samples tested negative in PCR analyses. These cases may be the result of low viral titre below the detection threshold of conventional PCR, uneven virus distribution in leaves sampled, or the presence of other begomoviruses [54,55,56]. Excluding these cases could lead to an underestimation of the test’s specificity and a misinterpretation of CMD prevalence. Future studies may be considered with complementary diagnostic approaches, such as alternative primers, other virus screening, and other diagnostic techniques (qPCR, NGS) to clarify the causes of symptomatic samples that are negative by conventional PCR [57].

We have identified the presence of two whitefly species, *Bemisia tabaci* and *Bemisia afer*, at the study sites. Both of them could potentially be responsible for propagating CMD in this site. While the involvement of *B. tabaci* in CMD transmission has been demonstrated [13,46,58,59,60], it is not the case for *B. afer*, which was exclusively found at the Bouaké and Dabou sites and was absent from the Man site. Similar observations were reported in an unpublished study by Amoakon et al. in Bouaké (399 m altitude) and Man (1050 m altitude). The study also did not reveal the presence of *B. afer* in the Man region. These observations suggest that the *B. afer* species is less prevalent in high-altitude regions. In contrast, the *B. tabaci* species was detected at all three study sites: Bouaké, Dabou, and Man. Despite some site-specific differences, *B. tabaci* biotypes (SSA1-SG1, SSA1-SG3, SSA1-SG5, and SSA3) were detected in both Bouaké and Man. In contrast, only SSA1-SG3, SSA1-SG5, and SSA3 were identified in Dabou. Among these, the SSA1-SG3 biotype was the most widespread across all three study sites. Furthermore, a greater diversity of whitefly species was observed in the low-altitude regions of Bouaké and Dabou compared to the high-altitude site of Man, aligning with the findings of Amoakon et al. [8]. Most of the whitefly species and biotypes identified in this study are known vectors of ACMV and EACMCMV, unlike *B. afer*, for which further investigations are urgently needed, given the very high populations found in cassava fields.

Given the alarming situation in Côte d’Ivoire, where CMD infection rates in cassava planting material exceed 90%, it is imperative to implement strict regulations governing the movement and distribution of cassava seeds. A practical and effective solution could be the establishment of a dedicated committee responsible for the sanitation, certification, management, and distribution of virus-free cassava cuttings in the country. This should be combined with a strong disease surveillance and rapid response system.

## 5. Conclusions

This work shows that the cassava varieties used in this study respond differently to cassava mosaic disease (CMD) depending on the agroecological zone. CMD symptoms appeared earlier at the Bouaké and Man sites (six weeks after planting) than at Dabou (eight weeks). However, infection levels were lower at the Man site. Whitefly populations were particularly high in Dabou, likely contributing to increased disease pressure. Although overall symptom severity remained relatively low across all sites, a significant positive correlation was observed between disease incidence, symptom severity, and whitefly abundance. Of all the plants initially used, only those from four resistant cassava varieties remained virus-free for the duration of the study in all the sites studied. The infection rate was influenced by several factors, including symptom severity, whitefly abundance, the specific study site, and the resistance level of each variety. Among the three sites, Man consistently produced the effective results, with the lowest CMD incidence, mildest symptoms, lowest whitefly populations, lowest infection rates, and least diversity of whitefly species. These characteristics make Man an ideal candidate region for the establishment of breeding programmes and healthy cassava cuttings. However, to maintain the phytosanitary quality of planting materials, regular monitoring and management of these fields will be essential. These results provide interesting insights while remaining objective. Further study using point methods over several years could support these findings.

## Figures and Tables

**Table 1:** Characteristics of the twelve cassava varieties used in this study [14].

Code	Variety Name	Type	Status of Resistance to Cassava Mosaic Disease
V1	Yacé CNRA	Local	Susceptible
V2	Boufouh4	Local	Tolerant
V3	TMS30572	Improved	Resistant
V4	Bayérè	Local	Susceptible
V5	TMS4(2)1425	Improved	Susceptible
V6	Yavo (TME7)	Improved	Susceptible
V7	Im89	Improved	Resistant
V8	Olékanga	Local	Tolerant
V9	Bocou 5 (TMS 98/0581)	Improved	Resistant
V10	Agbablé3	Local	Susceptible
V11	Bonoua 34	Local	Resistant
V12	Bocou 6 (M98/0068)	Improved	Resistant

**Table 2:** Description of scales used to assess the severity of cassava mosaic disease symptoms [31].

Scale	CMD Symptoms
1	Plants without symptoms
2	Plants showing moderate chlorotic spots or some deformation at the base
3	Plants with spots all over the leaf surface and twisting of the leaves
4	Plants with a leaf blade (up to 2/3 of the leaf surface) with a deformed leaf
5	Plants showing numerous symptoms of CMD and/or deformation, total loss of 4/5 of the leaf surface, and stunting of the entire plant

**Table 3:** Primer pairs used for begomovirus detection.

Primers	Sequences (5′ to 3′)	Target Region	Size	Reference
WAVE-508F	AAGGCCCATGTAAGGTCCAG	ACMV DNA-A AV1/AC3	800 pb	[15]
WAVE-1307R	GAAGGAGCTGGGGATTCACA			
ACMVB-F	TCGGGAGTGATACATGCGAAGGC	ACMV DNA-B (BV1/BC1)	628 pb	[34]
ACMVB-R	GGCTACACCAGCTACCTGAAGCT			
CMBRepF	CRT CAA TGA CGT TGT ACC A	EACMV DNA-A (AC1)	650 pb	[35]
EACMVRepR	GGT TTG CAG AGA ACT ACA TC			
WAVE-EB 1869F	TTCCAAGGGGAGGGTTCTGA	EACMV DNA-B (BC1)	800 pb	[15]
WAVE-EB 2694R	TGCTCTCGCCTCTCTCTTCT			
VNF031F	GGATACAGATAGGGTTCCCAC	EACMCMV DNA-A (AC2/AC3)	≈560 pb	[36]
VNF032R	GACGAGGACAAGAATTCCAAT			

**Table 4:** Relationship between CMBs, plant phytosanitary status, and CMD symptom severity.

Severity Level	Total Sample	Virus Detected			Negatives (%)
		ACMV (%)	EACMCMV (%)	ACMV + EACMCMV (%)	
Asymptomatic (score 1)	911	34 (3.73)	6 (0.66)	8 (0.88)	863 (94.73)
Moderate (score 2–3)	54	24 (44.44)	0 (0)	9 (16.67)	21 (38.89)
Severe (score 4)	41	22 (53.66)	0 (0)	11 (26.83)	8 (19.51)
Very severe (score 5)	5	2 (40)	0 (0)	3 (60)	0 (0)
Total	1011	82 (8.11)	6 (0.59)	31 (3.07)	892 (88.23)

## Data Availability

The raw data supporting the conclusions of this article will be made available by the authors on request.

## Supplementary Materials

The following supporting information can be downloaded at https://www.mdpi.com/article/10.3390/v17101393/s1, Supplementary Figure S1. Relationship between whitefly abundance per plant and (a–d) incidence of cassava mosaic disease (CMD) and (e–h) severity of CMD symptoms. Spearman’s rank correlation test was used to assess the relationships at a 5% significance level. Correlation strength was interpreted as follows: weak (0.1 < R < 0.3), moderate (0.3 < R < 0.5), and strong (0.5 < R < 0.7). Supplementary Figure S2. Dynamics of cassava mosaic begomovirus (CMB) infections by site and variety type: susceptible varieties (a–c), tolerant varieties (d–f), and resistant varieties (g–i). Supplementary Table S1. Mean of cassava mosaic disease (CMD) incidence, symptom severity, and whitefly abundance per plant, presented by experimental site and cassava variety. Supplementary Table S2. Percentage of detection of ACMV and EACMCMV begomoviruses. Supplementary Table S3. Percentage of infection according to the phytosanitary status of samples.

## References

[1] Rosenthal D.M., Ort D.R. (2012). Examining Cassava’s Potential to Enhance Food Security Under Climate Change. Trop. Plant Biol..

[2] FAOSTAT Food and Agriculture Organization of the United Nations Statistics. https://www.fao.org/faostat/fr/#data/QCL/visualize.

[3] Coulibaly O., Arinloye A.D., Faye M., Abdoulaye T., Calle-Goulivas A., Ahoyo R. (2014). Analyse des Chaines de Valeur Regionales du Manioc en Afrique de l’Ouest.

[4] Kouassi K.M., Mahyao A., N’zue B., Koffi E., Koffi C. (2018). Status of Cassava (*Manihot esculenta* Crantz) in Côte d’Ivoire: From Production to Consumption and Evaluation of Technology Adoption. Eur. Sci. J. ESJ.

[5] Vernier P., N’Zué B., Zakhia-Rozis N. (2018). Le Manioc, Entre Culture Alimentaire et Filière Agro-Industrielle.

[6] Zinga I., Chiroleu F., Valam Zango A., Ballot C.S.A., Harimalala M., Kosh Komba E., Yandia P.S., Semballa S., Reynaud B., Lefeuvre P. (2016). Evaluation of Cassava Cultivars for Resistance to Cassava Mosaic Disease and Yield Potential in Central African Republic. J. Phytopathol..

[7] ICTV (2024). Virus Taxonomy: 2024 Release.

[8] Amoakon W.J.-L., Yoboué A.A.N., Pita J.S., Mutuku J.M., N’Zué B., Combala M., Otron D.H., Koné M., Kouassi N.K., Sié R. (2023). Occurrence of Cassava Mosaic Begomoviruses in National Cassava Germplasm Preserved in Two Agro-ecological Zones of Ivory Coast. Plant Pathol..

[9] Kouakou B.S.M., Yoboué A.A.N., Pita J.S., Mutuku J.M., Otron D.H., Kouassi N.K., Kouassi K.M., Vanié-Léabo L.P.L., Ndougonna C., Zouzou M. (2024). Gradual Emergence of East African Cassava Mosaic Cameroon Virus in Cassava Farms in Côte d’Ivoire. Agronomy.

[10] Pita J.S., Fondong V.N., Sangaré A., Kokora R.N.N., Fauquet C.M. (2001). Genomic and Biological Diversity of the African Cassava Geminiviruses. Euphytica.

[11] Toualy M.N., Akinbade S., Koutoua S., Diallo H., Kumar P. (2014). Incidence and Distribution of Cassava Mosaic Begomoviruses in Côte d’Ivoire. Int. J. Agron. Agric. Res..

[12] Dubern J. (1994). Transmission of African Cassava Mosaic Geminivirus by the Whitefly (*Bemisia tabaci*). Trop. Sci..

[13] Romba R., Gnankine O., Drabo S.F., Tiendrebeogo F., Henri H., Mouton L., Vavre F. (2018). Abundance of *Bemisia tabaci* Gennadius (Hemiptera: Aleyrodidae) and Its Parasitoids on Vegetables and Cassava Plants in Burkina Faso (West Africa). Ecol. Evol..

[14] Amoakon W.J.-L., Combala M., Pita J.S., Mutuku J.M., N’Zué B., Otron D.H., Yéo E.F., Kouassi N.K., Sié R. (2023). Phenotypic Screening and Molecular Characterization of Cassava Mosaic Disease Resistance in Côte d’Ivoire Cassava Germplasm. Front. Sustain. Food Syst..

[15] Combala M., Pita J.S., Gbonamou M., Samura A.E., Amoakon W.J.-L., Kouakou B.S.M., Onile-ere O., Sawadogo S., Eboulem G.R., Otron D.H. (2024). An Alarming Eastward Front of Cassava Mosaic Disease in Development in West Africa. Viruses.

[16] Djaha K.E. (2019). Caractérisation Morphologique et Micropropagation des Cultivars de Manioc (*Manihot esculenta* Crantz) Assainis en Côte d’Ivoire. Ph.D. Thesis.

[17] Silué O., Kouadjo C.G.Z., Konan K.J.L., Konan A., Koffi K.E. (2022). Bien Produire In Vitro des Plants de Manioc Indemnes du Virus de la Mosaïque.

[18] Yéo E.F., Kouassi M.K., Pita J.S., Kouassi N.K., Koné D., N’guetta S.P.A. (2020). Using Thermotherapy And Meristem Tip Culture For Producing Virus-Free Cassava Planting Material From Six Varieties Cultivated In Côte d’Ivoire. Int. J. Sci. Technol..

[19] Benke A.P., Krishna R., Khandagale K., Gawande S., Shelke P., Dukare S., Dhumal S., Singh M., Mahajan V. (2023). Efficient Elimination of Viruses from Garlic Using a Combination of Shoot Meristem Culture, Thermotherapy, and Chemical Treatment. Pathogens.

[20] Akano A., Dixon A., Mba C., Barrera E., Fregene M. (2002). Genetic Mapping of a Dominant Gene Conferring Resistance to Cassava Mosaic Disease. Theor. Appl. Genet..

[21] Okorogri E.B., Adetimirin V.O., Ssemakula G., Odu B., Dixon A.G.O. (2010). Rate of Re-Infection of Tissue Culture-Derived Latin American and East and Southern African Cassava Genotypes by Mosaic Disease. Afr. J. Biotechnol..

[22] Bairu M.W., Amoo S.O., Van Staden J. (2011). Comparative Phytochemical Analysis of Wild and in Vitro-Derived Greenhouse-Grown Tubers, in Vitro Shoots and Callus-like Basal Tissues of *Harpagophytum procumbens*. S. Afr. J. Bot..

[23] Beyene G., Chauhan R.D., Wagaba H., Moll T., Alicai T., Miano D., Carrington J.C., Taylor N.J. (2016). Loss of CMD2-Mediated Resistance to Cassava Mosaic Disease in Plants Regenerated through Somatic Embryogenesis. Mol. Plant Pathol..

[24] Chauhan R.D., Beyene G., Taylor N.J. (2018). Multiple Morphogenic Culture Systems Cause Loss of Resistance to Cassava Mosaic Disease. BMC Plant Biol..

[25] Dibi K.E.B., Essis B.S., Kouamé K.T., Essy A.R.-F., Kouakou A.M., Dogbo D.O., N’Zue B. (2024). Effet de la Durée de Conservation des Boutures sur le Rendement et la Qualité des Racines Tubéreuses du Manioc (*Manihot esculenta* Crantz) au Centre de la Côte d’Ivoire. Tropicultura.

[26] Doubi B.T.S., Kouassi K.I., Kouakou K.L., Koffi K.K., Baudoin J.-P., Zoro B.I.A. (2016). Existing Competitive Indices in the Intercropping System of *Manihot esculenta* Crantz and *Lagenaria siceraria* (Molina) Standley. J. Plant Interact..

[27] N’zué B., Zouhouri P.G., Sangaré A. (2004). Performances Agronomiques de Quelques Varietes de Manioc (*Manihot esculenta* Crantz) Dans Trois Zones Agroclimatiques de la Cote D’Ivoire. Agron. Afr..

[28] Boni N., Pamelas O.M., Michel K.A., Brice D.K.E., Pierre Z.G., Sidoine E.B., Alexandre D.A. (2012). Morphological Characterization of Cassava (*Manihot esculenta* Crantz) Accessions Collected in the Centre-West, South-West and West of Côte d’Ivoire. Greener J. Agric. Sci..

[29] Seka J.S.S., Kouassi M.K., Yéo E.F., Saki F.M., Otron D.H., Tiendrébéogo F., Eni A., Kouassi N.K., Pita J.S. (2025). Removing Recalcitrance to the Micropropagation of Five Farmer-Preferred Cassava Varieties in Côte d’Ivoire by Supplementing Culture Medium with Kinetin or Thidiazuron. Front. Plant Sci..

[30] Akano A.O., Atiri G.I., Ng S.Y.C., Asiedu R. (1997). Effect of African Cassava Mosaic Disease on Growth and Yield Components of Virus-Tested Cassava Genotypes Derived from Meristem Culture in Early and Late Planting Periods in Three Agroecologies of Nigeria. Afr. J. Root Tuber. Crops.

[31] Hahn S.K., Terry E.R., Leuschner K. (1980). Breeding Cassava for Resistance to Cassava Mosaic Disease. Euphytica.

[32] Sseruwagi P., Sserubombwe W.S., Legg J.P., Ndunguru J., Thresh J.M. (2004). Methods of Surveying the Incidence and Severity of Cassava Mosaic Disease and Whitefly Vector Populations on Cassava in Africa: A Review. Virus Res..

[33] Doyle J.J., Doyle J.L. (1987). A Rapid DNA Isolation Procedure for Small Quantities of Fresh Leaf Tissue. Phytochem. Bull..

[34] Matic S., da Cunha A.P., Thompson J.R., Tepfer M. (2012). An Analysis of Viruses Associated with Cassava Mosaic Disease in Three Angolan Provinces. J. Plant Pathol..

[35] Alabi O.J., Ogbe F.O., Bandyopadhyay R., Lava Kumar P., Dixon A.G., Hughes J.A., Naidu R.A. (2008). Alternate Hosts of African Cassava Mosaic Virus and East African Cassava Mosaic Cameroon Virus in Nigeria. Arch. Virol..

[36] Fondong V.N., Pita J.S., Rey M.E.C., de Kochko A., Beachy R.N., Fauquet C.M. (2000). Evidence of Synergism between African Cassava Mosaic Virus and a New Double-Recombinant Geminivirus Infecting Cassava in Cameroon. J. General. Virol..

[37] Mugerwa H., Seal S., Wang H.-L., Patel M.V., Kabaalu R., Omongo C.A., Alicai T., Tairo F., Ndunguru J., Sseruwagi P. (2018). African Ancestry of New World, *Bemisia tabaci*-Whitefly Species. Sci. Rep..

[38] Kumar S., Stecher G., Li M., Knyaz C., Tamura K. (2018). MEGA X: Molecular Evolutionary Genetics Analysis across Computing Platforms. Mol. Biol. Evol..

[39] Wickham H. (2016). Data Analysis. ggplot2.

[40] Venables W.N., Smith D., R Core Team (2024). An Introduction to R-Notes on R: A Programming Environment for Data Analysis and Graphics.

[41] Patil B.L., Fauquet C.M. (2009). Cassava Mosaic Geminiviruses: Actual Knowledge and Perspectives. Mol. Plant Pathol..

[42] FAO (2014). FAO Statistical Yearbook 2014 Africa Food and Agriculture. Proceedings of the 2016 ASABE Annual International Meeting.

[43] Otim-Nape G.W., Thresh J.M., Jones D.G. (1998). The Current Pandemic of Cassava Mosaic Virus Disease in Uganda. The Epidemiology of Plant Diseases.

[44] Harimalala M., Chiroleu F., Giraud-Carrier C., Hoareau M., Zinga I., Randriamampianina J.A., Velombola S., Ranomenjanahary S., Andrianjaka A., Reynaud B. (2015). Molecular Epidemiology of Cassava Mosaic Disease in Madagascar. Plant Pathol..

[45] Hasanuzzaman A.T.M., Islam M.N., Zhang Y., Zhang C.-Y., Liu T.-X. (2016). Leaf Morphological Characters Can Be a Factor for Intra-Varietal Preference of Whitefly *Bemisia tabaci* (Hemiptera: Aleyrodidae) among Eggplant Varieties. PLoS ONE.

[46] Prado J.C., Peñaflor M.F.G.V., Cia E., Vieira S.S., Silva K.I., Carlini-Garcia L.A., Lourenção A.L. (2016). Resistance of Cotton Genotypes with Different Leaf Colour and Trichome Density to *Bemisia tabaci* Biotype B. J. Appl. Entomol..

[47] Silva M.S., Lourenção A.L., Souza-Dias J.A.C., Miranda Filho H.S., Ramos V.J., Schammass E.A. (2008). Resistance of Potato Genotypes (*Solanum* spp.) to *Bemisia tabaci* Biotype B. Hortic. Bras..

[48] Morales F.J., Jones P.G. (2004). The Ecology and Epidemiology of Whitefly-Transmitted Viruses in Latin America. Virus Res..

[49] Baulcombe D.C. (1996). RNA as a Target and an Initiator of Post-Transcriptional Gene Silencing in Trangenic Plants. Post-Transcriptional Control of Gene Expression in Plants.

[50] Napoli C., Lemieux C., Jorgensen R. (1990). Introduction of a Chimeric Chalcone Synthase Gene into Petunia Results in Reversible Co-Suppression of Homologous Genes in Trans. Plant Cell.

[51] Waterhouse P.M., Wang M.-B., Lough T. (2001). Gene Silencing as an Adaptive Defence against Viruses. Nature.

[52] Vanitharani R., Chellappan P., Pita J.S., Fauquet C.M. (2004). Differential Roles of AC2 and AC4 of Cassava Geminiviruses in Mediating Synergism and Suppression of Posttranscriptional Gene Silencing. J. Virol..

[53] Chellappan P., Vanitharani R., Fauquet C.M. (2004). Short Interfering RNA Accumulation Correlates with Host Recovery in DNA Virus-Infected Hosts, and Gene Silencing Targets Specific Viral Sequences. J. Virol..

[54] Zhang Y., Wei Z., Zhang J., Chen C., Liu F. (2025). Application of PCR and PCR-Derived Technologies for the Detection of Pathogens Infecting Crops. Physiol. Mol. Plant Pathol..

[55] Allado S.S., Adjata D.K., Pita J.S., Mivedor A.S., Dansou-Kodjo K.A., Tozo K. (2023). Multiplex PCR for Identification and Detection of Cassava Mosaic Begomoviruses in Togo. Adv. Microbiol..

[56] Boonham N., Kreuze J., Winter S., van der Vlugt R., Bergervoet J., Tomlinson J., Mumford R. (2014). Methods in Virus Diagnostics: From ELISA to next Generation Sequencing. Virus Res..

[57] Otron D.H., Filloux D., Brousse A., Hoareau M., Fenelon B., Hoareau C., Fernandez E., Tiendrébéogo F., Lett J.-M., Pita J.S. (2025). Improvement of Nanopore Sequencing Provides Access to High Quality Genomic Data for Multi-Component CRESS-DNA Plant Viruses. Virol. J..

[58] Berry S.D., Fondong V.N., Rey C., Rogan D., Fauquet C.M., Brown J.K. (2004). Molecular Evidence for Five Distinct *Bemisia tabaci* (Homoptera: Aleyrodidae) Geographic Haplotypes Associated with Cassava Plants in Sub-Saharan Africa. Ann. Entomol. Soc. Am..

[59] Nwezeobi J., Onyegbule O., Nkere C., Onyeka J., Brunschot S., Seal S., Colvin J. (2020). Cassava Whitefly Species in Eastern Nigeria and the Threat of Vector-Borne Pandemics from East and Central Africa. PLoS ONE.

[60] Efekemo O.P., Onile-ere O.A., Abegunde I.O., Otitolaye F.T., Pita J.S., Alicai T., Eni A.O. (2024). Molecular Diversity and Distribution of Whiteflies (*Bemisia tabaci*) in Cassava Fields Across South West and North Central, Nigeria. Insects.

