# Supplementary material for: Reinfection Dynamics of Disease-Free Cassava Plants in Three Agroecological Regions of Côte d’Ivoire
Source: Viruses. 2025 Oct 20;17(10):1393. doi: 10.3390/v17101393 (PMC12567664; doi:10.3390/v17101393)
Supplement: Supplementary file 1 [file viruses-17-01393-s001.zip › viruses-3904490-supplementary.pdf]

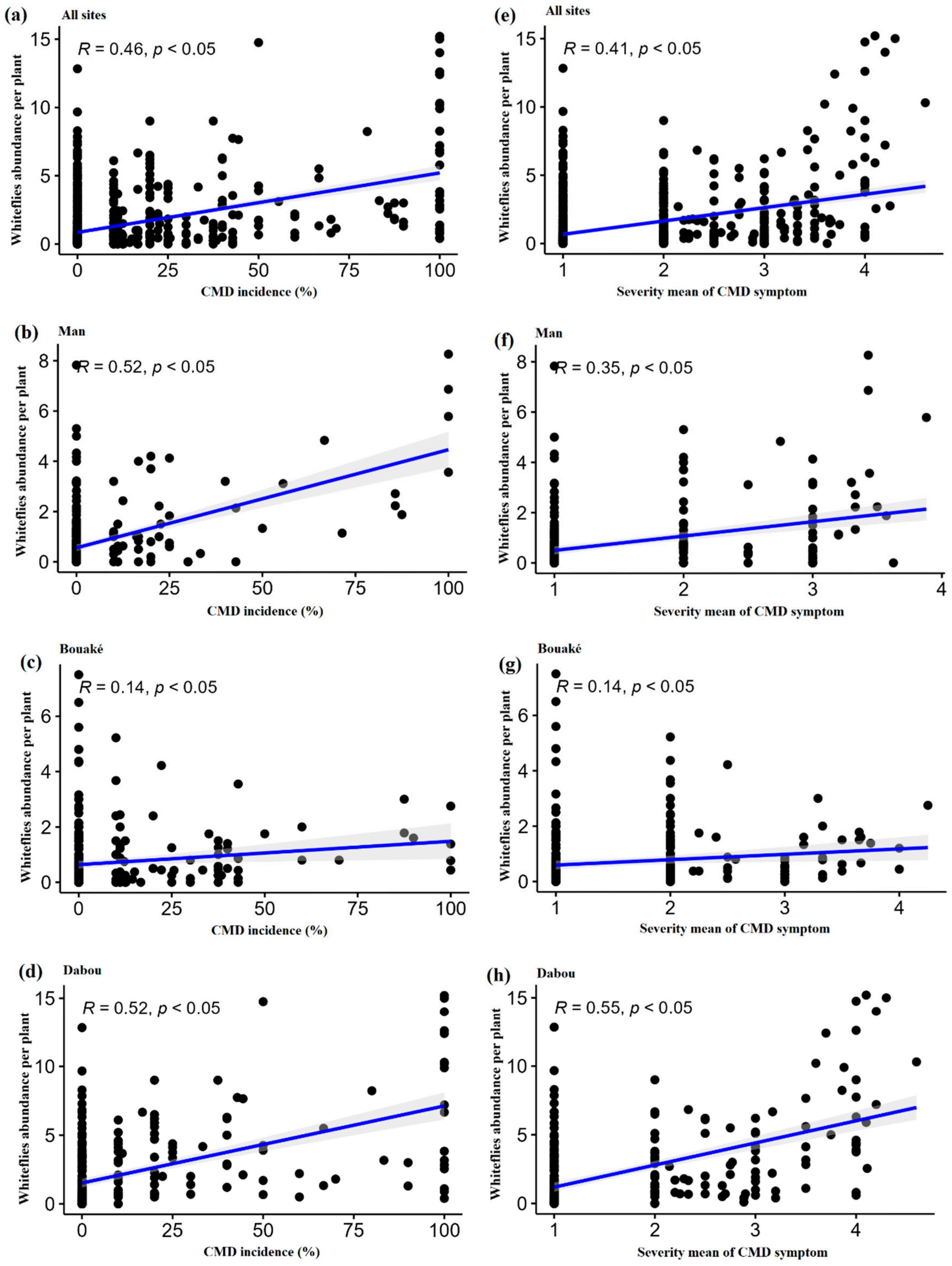

Supplementary Figure S1. Relationship between whitefly abundance per plant and (a–d) incidence of cassava mosaic disease (CMD) and (e–h) severity of CMD symptoms. Spearman's rank correlation test was used to assess the relationships at a 5% significance level. Correlation strength was interpreted as follows: weak $(0.1 < R < 0.3)$, moderate $(0.3 < R < 0.5)$, and strong $(0.5 < R < 0.7)$.

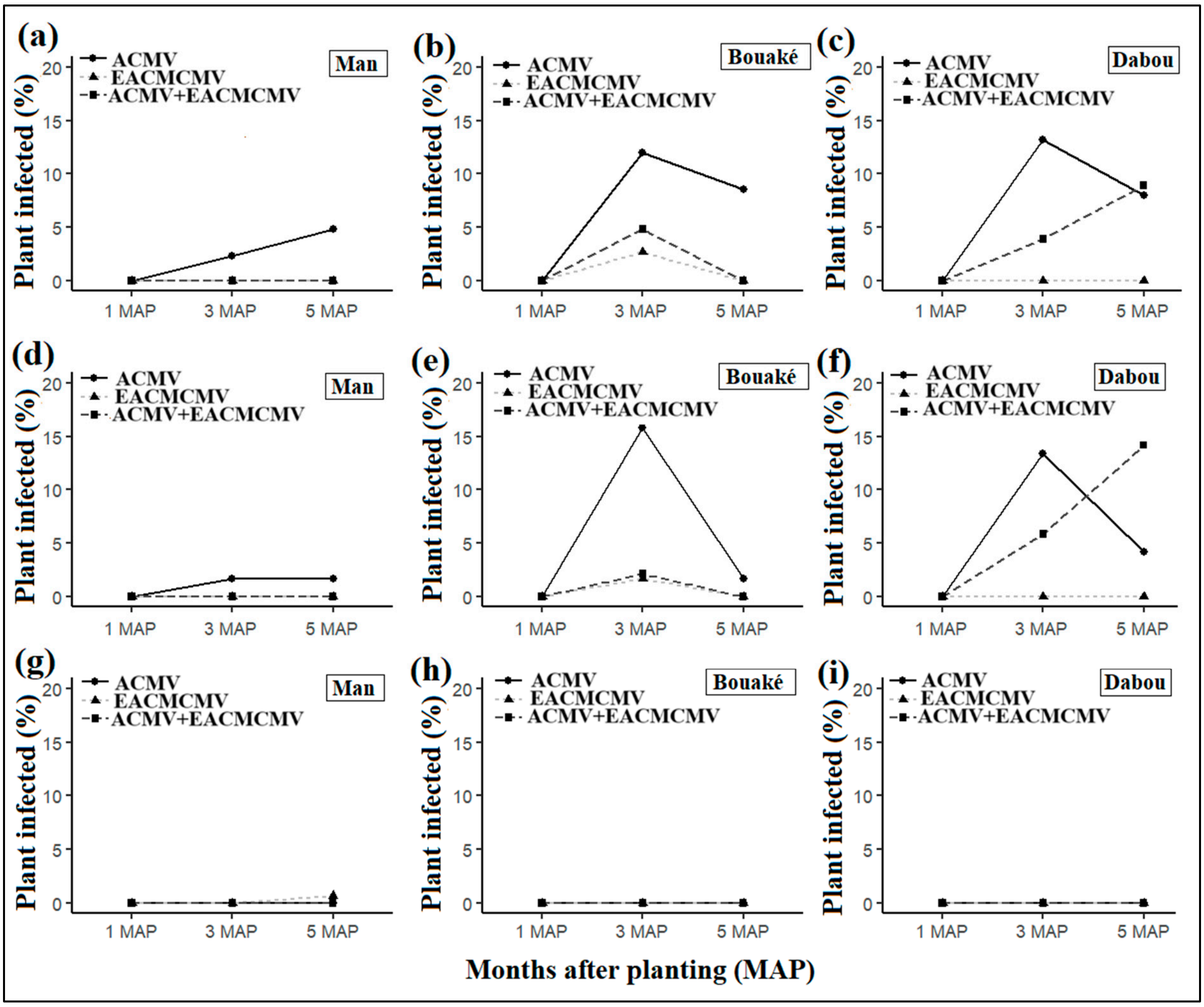

Supplementary Figure S2. Dynamics of cassava mosaic begomovirus (CMB) infections by site and variety type: susceptible varieties (a–c), tolerant varieties (d–f), and resistant varieties (g–i).

Supplementary Table S1. Mean of cassava mosaic disease (CMD) incidence, symptom severity, and whitefly abundance per plant, presented by experimental site and cassava variety. Values are means ± standard error. Within each column. means followed by the same letter are not significantly different for each variety according to Tukey-adjusted *emmeans* post-hoc test at the 5% level. V1: Yacé; V2: Boufouh 4; V3: TMS30572; V4: Bayérè; V5: TMS4(2)1425; V6: Yavo; V7: IM89; V8: Olékanga; V9: Bocou 5; V10: Agbablé 3; V11: Bonoua 34; V12: Bocou 6.

Varieties	Sites	N	CMD incidence (%)	Severity means of CMD symptoms	Whitefly abundance per plant
	Bouaké	24	4.04 ± 1.77b	2.75 ± 0.17a	0.22 ± 0.06b
V1	Dabou	24	22.5 ± 7a	2.41 ± 0.11b	0.96 ± 23a
	Man	24	0.93 ± 0.09b	2 ± 0b	0.27 ± 0.18b
	Bouaké	24	5.38 ± 1.65b	2.21 ± 0.09b	0.94 ± 0.24b
V2	Dabou	24	8.33 ± 2.6a	2.65 ± 0.21a	2.3 ± 0.47a
	Man	24	4.17 ± 1.46b	2.19 ± 0.09b	1.07 ± 0.28b

V3	Bouaké	24	0 ± 0a	1 ± 0a	0.36 ± 0.11b
	Dabou	24	0 ± 0a	1 ± 0a	1.08 ± 0.22a
	Man	24	0 ± 0a	1 ± 0a	0.37 ± 0.1b
V4	Bouaké	24	24.63 ± 3.57a	2.33 ± 0.11b	0.3 ± 0.08b
	Dabou	24	24.64 ± 5.69a	3.16 ± 0.2a	3.41 ± 0.79a
	Man	24	3.17 ± 1.65b	2.17 ± 0.11b	0.6 ± 0.22b
V5	Bouaké	24	8.09 ± 3.11ab	2.17 ± 0.09a	0.51 ± 0.16b
	Dabou	24	9.21 ± 2.46a	2.3 ± 0.15a	1.59 ± 0.37a
	Man	24	5.16 ± 2.01b	2.21 ± 0.11a	0.75 ± 0.21b
V6	Bouaké	24	42.64 ± 7.99b	2.82 ± 0.17b	1.13 ± 0.25b
	Dabou	24	52.92 ± 9.47a	3.47 ± 0.22a	4.89 ± 1.18a
	Man	24	37.9 ± 8.45b	2.76 ± 0.17b	1.93 ± 0.46b
V7	Bouaké	24	0 ± 0a	1 ± 0a	0.77 ± 0.28a
	Dabou	24	0 ± 0a	1 ± 0a	1.1 ± 0.27a
	Man	24	0 ± 0a	1 ± 0a	0.32 ± 0.12a
V8	Bouaké	24	8.24 ± 2.1ab	2.14 ± 0.1b	0.96 ± 0.26b
	Dabou	24	10.42 ± 2.52a	2.75 ± 0.21a	2.29 ± 0.49a
	Man	24	6.77 ± 2.39b	2.11 ± 0.06b	0.9 ± 0.27b
V9	Bouaké	24	0 ± 0a	1 ± 0a	1.61 ± 0.42b
	Dabou	24	0 ± 0a	1 ± 0a	4.07 ± 0.71a
	Man	24	0 ± 0a	1 ± 0a	1.44 ± 0.36b
V10	Bouaké	24	9.56 ± 3.67b	2.51 ± 0.18a	0.61 ± 0.14b
	Dabou	24	29.86 ± 8.33a	2.79 ± 0.14a	2.29 ± 0.48a
	Man	24	13.73 ± 3.71b	2.18 ± 0.09b	0.85 ± 0.27b
V11	Bouaké	24	0 ± 0a	1 ± 0a	0.56 ± 0.21a
	Dabou	24	0 ± 0a	1 ± 0a	1.25 ± 0.25a
	Man	23	0 ± 0a	1 ± 0a	0.61 ± 0.2a
V12	Bouaké	24	0 ± 0a	1 ± 0a	0.53 ± 0.16b
	Dabou	23	0 ± 0a	1 ± 0a	1.81 ± 0.41a
	Man	25	0 ± 0a	1 ± 0a	0.39 ± 0.1b

Supplementary Table S2. Percentage of detection of ACMV and EACMCMV begomoviruses

**Total number of samples tested**	**Virus detected**				**Negative**
	**ACMV**	**EACMV**	**EACMCMV**	**ACMV+EACMCMV**	
1011 (100 %)	100 (9. 89 %)	0 (0%)	3 (0.3 %)	28 (2.77 %)	890 (87.04 %)

Supplementary Table S3. Percentage of infection according to the phytosanitary status of samples

	**Symptomatic**	**Asymptomatic**
**Total number of samples tested**	100 (100 %)	911 (100 %)
**ACMV**	48 (48 %)	34 (3.72 %)
**EACMCMV**	0 (0 %)	6 (0.66 %)
**ACMV+EACMCMV**	23 (23 %)	8 (0.88 %)
**Negatives PCR**	29 (29 %)	863 (94.73 %)
